# *De novo* assembly and comparative analysis of the first complete mitochondrial genome of the genus *Anisomeles* (*Anisomeles indica*)

**DOI:** 10.3389/fpls.2026.1982031

**Published:** 2026-10-21

**Authors:** Xiaogang Dai, Xiaofan Cai, Yueshan Fu, Shan Wang, Changwei Bi

**Affiliations:** 1 State Key Laboratory for Tree Genetics and Breeding, Co-Innovation Center for Sustainable Forestry in Southern China, Key Laboratory of Tree Genetics and Biotechnology of Educational Department of China, Jiangsu Key Laboratory of Tree Biological Breeding and Seed Industry, Nanjing Forestry University, Nanjing, China; 2 College of Information Science and Technology & Artificial Intelligence, Nanjing Forestry University, Nanjing, China; 3 Jiangsu Engineering and Technology Center for Modern Horticulture, Jiangsu Vocational College of Agriculture and Forestry, Zhenjiang, China

**Keywords:** *Anisomeles indica*, comparative analysis, Lamiaceae, mitochondrial genome, phylogenetic analysis

## Abstract

*Anisomeles indica* (L.) Kuntze is an important traditional medicinal plant with diverse pharmacological activities, while no mitochondrial genome (mitogenome) has yet been reported for this species or any member of the genus, limiting our understanding of organelle genome evolution and molecular characteristics within Lamiaceae. In this study, we report the first complete mitogenome of the genus *Anisomeles*, which was *de novo* assembled and annotated for *A. indica* using PacBio high-fidelity (HiFi) sequencing data. The mitogenome of *A. indica* was assembled as a single master circular molecule with a length of 296,457 bp and a GC content of 45.42%. A total of 51 genes were identified, including 32 protein-coding genes (PCGs), three rRNA genes and 16 tRNA genes. Repeat analysis revealed relatively low repeat abundance, with 60 simple sequence repeats, 136 dispersed repeats, and 15 tandem repeats identified. Furthermore, 30 mitochondrial plastid DNA sequences were detected (24,696 bp), suggesting a substantial contribution of plastid-derived sequences to the mitogenome. RNA editing analysis predicted 444 editing sites across all mitochondrial PCGs except *atp1*. Phylogenetic analyses based on mitochondrial and plastid genome datasets supported a closer relationship of *A. indica* with *Pogostemon* than with *Phlomoides*. This study provides the first mitogenomic resource for *Anisomeles* and establishes a foundation for future studies of mitogenome evolution, comparative genomics, and phylogenetic relationships within Lamiaceae.

## 1 Introduction

*Anisomeles indica* (L.) Kuntze is a medicinal herb belonging to the genus *Anisomeles* R. Br. in the family Lamiaceae. The genus comprises approximately seven to eight herbaceous species distributed mainly in tropical and subtropical regions of Asia (Li et al., 2016; Yao et al., 2016). Among them, *A. indica* has long been used as an important medicinal plant in traditional Chinese medicine (Yao et al., 2019). The whole plant is utilized as herbal medicine and is described as possessing pungent and bitter properties (Kunwar et al., 2010). Traditionally, *A. indica* has been used for managing diverse health conditions, including inflammatory skin diseases, gastrointestinal disorders, liver-related diseases and rheumatic ailments (Rao et al., 2009). In addition to its medicinal value, phytochemical investigations have demonstrated that *A. indica* contains abundant bioactive constituents, including terpenoids, flavonoids, phenolic compounds, and essential oils, many of which exhibit anti-inflammatory, antioxidant, antimicrobial, hepatoprotective, and antitumor activities, highlighting its considerable medicinal and pharmacological potential (Chen et al., 2008, 2023). For example, ovatodiolide, a macrocyclic diterpenoid isolated from *A. indica*, has demonstrated diverse pharmacological activities, including anticancer, antiviral, anti-inflammatory, and antifibrotic effects, further supporting the biomedical importance of this species (Wu et al., 2020; Yu et al., 2022; Zhou et al., 2026).

As semi-autonomous organelles responsible for cellular respiration and energy production, mitochondria possess their own genetic systems and exhibit evolutionary patterns that differ markedly from those of nuclear and plastid genomes (Kummer and Ban, 2021; Moura et al., 2025). Plant mitochondrial genomes (mitogenomes) have attracted considerable attention because they provide important insights into organelle genome evolution, intracellular DNA transfer and phylogenetic relationships, while also serving as valuable genetic resources for crop improvement and molecular breeding (Gray et al., 1999; Dyall et al., 2004). Unlike the relatively conserved plastid genomes (plastomes), plant mitogenomes are among the most structurally dynamic genomes in eukaryotes. They exhibit remarkable variation in genome size, ranging from approximately 66 kb in *Viscum scurruloideum* to 18.99 Mb in *Cathaya argyrophylla* (Skippington et al., 2015; Huang et al., 2025).

The structural complexity of plant mitogenomes has posed substantial challenges for genome assembly and annotation (Wang et al., 2025b). Large repeat sequences (repeats), alternative genome conformations, and the frequent incorporation of foreign DNA, particularly mitochondrial plastid DNA sequences (MTPTs), greatly increase assembly difficulty even with the development of long-read sequencing technologies (Park et al., 2020; Nhat Nam et al., 2024). Nuclear mitochondrial DNA sequences (NUMTs) can further confound the assignment of reads to the mitogenome. Consequently, the number of publicly available complete plant mitogenomes remains far lower than that of plastomes. According to the Plant Mitochondrial Genome Database (PMGD; http://www.pmgdb.cn/), approximately 1,830 complete plant mitogenomes have been reported (Yang et al., 2026), whereas more than 42,000 plastomes are currently available on the Chloroplast Genome Information Resource website (CGIR; https://ngdc.cncb.ac.cn/cgir/). Expanding taxonomic sampling of plant mitogenomes is therefore essential for improving our understanding of mitogenome evolution and diversity across angiosperms.

Advances in sequencing technologies have greatly accelerated the accumulation of published plant mitogenomes, yet mitochondrial genomic resources available for Lamiaceae are still relatively scarce. We found that no more than 50 complete mitogenomes from Lamiaceae species were publicly available in the NCBI database (last accessed on 6 August 2026), representing a small fraction of the family’s taxonomic diversity. Moreover, no complete mitogenome has yet been reported for any species of the genus *Anisomeles*, leaving a significant gap in our understanding of mitogenome evolution within this lineage of Lamiaceae. Among the members of this genus, *A. indica* has attracted increasing attention in studies of phytochemistry, pharmacology, and molecular biology (Rao et al., 2009; Nasrin et al., 2022; Zhou et al., 2026). Although its complete plastome has been reported, the mitogenome of *A. indica* has not previously been characterized in a published study (Yao et al., 2019). Therefore, the characterization of the first *Anisomeles* mitogenome is crucial for expanding mitochondrial genomic resources within Lamiaceae and will facilitate a better understanding of mitogenome evolution, structural variation, and phylogenetic relationships across this economically and ecologically important family.

In this study, we successfully assembled and annotated the complete mitogenome of *A. indica*, the first mitogenome for the genus *Anisomeles*. We comprehensively analyzed its genomic features, codon usage, repeats, MTPTs and RNA editing events. To examine mitogenome diversity within Lamiaceae, we compiled a comparative dataset of 42 mitogenomes, comprising the newly assembled *A. indica* mitogenome and 41 reference mitogenomes retrieved from NCBI (**Supplementary Table S1**). We then conducted comparative analyses and reconstructed phylogenetic relationships with representative members of Lamiaceae. This study will expand the current knowledge of mitogenome evolution in Lamiaceae and provide valuable genetic resources for future studies on organelle genome evolution, phylogenetics, and the conservation and utilization of this medicinal species.

## 2 Materials and methods

### 2.1 Plant materials, DNA extraction and sequencing

Fresh leaves of *A. indica* were collected by Xiaogang Dai in October 2025 from the Baima Campus of Nanjing Forestry University, Nanjing, Jiangsu, China (31.61° N, 119.18° E) and immediately frozen in liquid nitrogen before storage at −80 °C. The taxonomic identity of the collected material was confirmed by a botanist based on morphological characteristics, and no herbarium voucher was deposited for the material used in this study. High-quality total genomic DNA was extracted from fresh leaves using the cetyltrimethylammonium bromide (CTAB) method (Arseneau et al., 2017). DNA quality and concentration were assessed using a NanoDrop 2000 spectrophotometer (Thermo Fisher Scientific, USA) and 1.0% agarose gel electrophoresis. Sequencing libraries were constructed using the SMRTbell Express Template Prep Kit 2.0 (Pacific Biosciences, Inc., CA, USA). PacBio HiFi sequencing was performed using the PacBio Revio sequencing system (Pacific Biosciences, Inc., CA, USA) by Haorui Genomics (Xi’an, Shaanxi, China). A total of 5,210,225 HiFi reads comprising 82.48 Gb of sequence data were generated.

### 2.2 Mitogenome assembly and annotation

For mitogenome assembly, 20% of the total HiFi dataset was used according to the PMAT parameter “-F 0.2”, corresponding to 1,042,052 reads with a total length of 16.50 Gb and a mean read length of 15,829.4 bp. The selected HiFi reads were assembled using PMAT v2.0 with the parameters “-t hifi -m -F 0.2 -T 50” under the “autoMito” mode (Bi et al., 2024a). The assembly graph was manually inspected and resolved using Bandage (Wick et al., 2015), yielding a complete circular mitogenome of 296,457 bp with an estimated mean sequencing depth of 209.3×, calculated after excluding two plastid-homologous sequences from the coverage analysis. To further evaluate the assembly topology, read-level validation was performed for the alternative connections associated with the 247-bp repetitive node R1997 in the assembly graph. Candidate junction sequences representing the master-circle configuration (407→R1997→785 and 785→2194→R1997→423) and the alternative two-circle configuration (407→R1997→423 and 785→2194→R1997→785) were constructed separately. PacBio HiFi reads were remapped to each candidate junction sequence using minimap2 v2.31-r1302 with the map-hifi preset (Li, 2018). To minimize false support from reads mapping only within the repetitive region, only reads that continuously spanned R1997 together with 2 kb of unique flanking sequence on each side and showed an alignment identity of ≥99% were retained as junction-supporting reads. The resulting read alignments across the diagnostic junctions were visually inspected using Integrative Genomics Viewer (IGV) v2.19.8 software (Robinson et al., 2011). In addition, the HiFi reads were independently assembled using OATK (Zhou et al., 2025). The OATK assembly yielded a single circular mitogenome of 296,460 bp, which was highly consistent with the 296,457-bp mitogenome obtained using PMAT. Genome annotation was performed using the online program PMGA (http://www.1kmpg.cn/pmga/) against its built-in reference database containing 319 annotated angiosperm mitochondrial genomes (Li et al., 2025). Protein-coding genes (PCGs) were manually checked, whereas rRNA genes (rRNAs) and tRNA genes (tRNAs) were verified using BLASTn and tRNAscan-SE v2.0 (Camacho et al., 2009; Chan et al., 2021), respectively. The circular mitogenome map was finally generated using the PMGmap program with default parameters (Zhang et al., 2024).

### 2.3 Repeat identification and codon usage analysis

Simple sequence repeats (SSRs) were identified using the MISA program with minimum repeat thresholds of “10, 5, 4, 3, 3, and 3” repeat units for “mono-, di-, tri-, tetra-, penta-, and hexanucleotide” motifs, respectively (Thiel et al., 2003; Beier et al., 2017). Dispersed repeats were detected using Vmatch v2.3.1 with the parameters “-d -p -h 3 -l 30 -best 5000 -noscore -noidentity -absolute” (Kurtz, 2011). Tandem repeats were identified using Tandem Repeats Finder v4.09 with default settings (Benson, 1999). The repeat characteristics of *A. indica* were further compared with those of three representative Lamiaceae species (*Phlomoides rotata*, *Rotheca serrata* and *Scutellaria barbata*) using the same parameters. For the family-wide comparison, a dataset comprising the *A. indica* mitogenome and 41 reference Lamiaceae mitogenomes was analyzed to evaluate the characteristics of repetitive elements within the family (**Supplementary Table S1**). The circumscription of Lamiaceae followed Li et al. (2016). For multichromosomal mitogenomes, genome size and GC content were summarized across all constituent sequence records. SSRs, dispersed repeats, and tandem repeats were identified across this dataset using the same parameters. As for codon usage, relative synonymous codon usage (RSCU) values for all PCGs were calculated using custom Python scripts developed in-house.

### 2.4 RNA editing prediction and experimental validation

Potential RNA editing sites in mitochondrial PCGs were predicted using Deepred-mt (Edera et al., 2021). Only predicted editing sites with probabilities ≥0.90 were retained for subsequent analyses. To experimentally validate representative predicted RNA editing sites, total RNA and genomic DNA were extracted from the sampled plant material. Complementary DNA (cDNA) was synthesized from total RNA, and selected regions of *nad4*, *mttB*, and *rps4* were amplified by reverse transcription PCR (RT-PCR) using gene-specific primers (**Supplementary Table S2**). The corresponding genomic DNA regions were amplified in parallel using the same primer pairs. The resulting PCR products were subjected to Sanger sequencing, and the genomic DNA and cDNA sequences were compared to identify C-to-U RNA editing events.

### 2.5 MTPT identification

To identify MTPT sequences, the complete plastome of *A. indica* (GenBank accession number: NC_046781) was downloaded from the NCBI database. Homologous regions between the mitogenome and plastome of *A. indica* were detected using BLASTn with the parameters “-word_size 9 -evalue 1e-5 -reward 2 -gapopen 5 -gapextend 2 -penalty -3 -outfmt 6” (Camacho et al., 2009). Alignments with sequence identity ≥70% and length ≥100 bp were retained and manually curated. The distribution of MTPT fragments was visualized using the Python package pyCircos.

### 2.6 Synteny analysis

To investigate mitogenome organization and structural variation within Lamiaceae, four representative Lamiaceae mitogenomes (*P. rotata*, *Pogostemon heyneanus*, *R. serrata* and *S. barbata*) were obtained from the NCBI database for comparative analysis with the newly assembled mitogenome of *A. indica*. Syntenic blocks were identified based on BLASTn alignments with a minimum alignment length of 200 bp and sequence identity ≥80%. The identified syntenic regions were visualized using NGenomeSyn (He et al., 2023).

### 2.7 Phylogenetic analysis

Complete mitogenome and plastome sequences of representative Lamiales species were downloaded from the NCBI Nucleotide database. Because no complete plastome sequence is currently available for *P. heyneanus*, different representatives of the genus *Pogostemon* were included in the two organellar datasets: *P. heyneanus* was used in the mitogenome dataset, whereas *Pogostemon septentrionalis* was selected as the representative species of *Pogostemon* in the plastome dataset. *Capsicum annuum* and *Nicotiana tabacum* (Solanaceae) were selected as outgroups. Detailed information on all mitogenomes and plastomes used in phylogenetic analysis and corresponding accession numbers is provided in **Supplementary Table S3**. For phylogenetic reconstruction, 26 conserved mitochondrial PCGs and 52 shared plastid PCGs were extracted from the corresponding datasets. Each gene was independently aligned using MUSCLE v5 (Edgar, 2022), and the aligned sequences were concatenated using custom Python scripts. Maximum-likelihood (ML) phylogenetic trees were reconstructed using IQ-TREE v3.1.2 with the parameters “-m MFP --ufboot 1000” (Kalyaanamoorthy et al., 2017; Hoang et al., 2018; Wong et al., 2026). The best-fit nucleotide substitution models were selected according to the Bayesian Information Criterion, with “TVM+F+I+R3” selected for the mitogenome dataset and “GTR+F+R3” selected for the plastome dataset. The resulting phylogenetic trees were visualized using the Interactive Tree Of Life (iTOL; https://itol.embl.de/) (Letunic and Bork, 2024).

## 3 Results

### 3.1 Characteristics of the *A. indica* mitogenome

The complete mitogenome of *A. indica* was assembled as a single master circular molecule after manual resolution of the assembly graph using Bandage (**Figures 1A, B**). The assembled genome is 296,457 bp in length and has an overall GC content of 45.42% (**Figure 1C**; **Table 1**). Read-level validation of the alternative connections around the repetitive node R1997 showed that the two diagnostic junctions of the master-circle configuration were supported by 167 and 145 HiFi reads, respectively, whereas only 5 and 5 reads supported the corresponding junctions of the alternative two-circle configuration (**Supplementary Figure S1**). Independent assembly using OATK also recovered a single circular mitogenome of 296,460 bp, differing by only 3 bp from the PMAT assembly. A total of 51 genes were annotated, collectively spanning 35,891 bp (12.11% of the mitogenome). These comprise 32 PCGs (24 core and eight variable mitochondrial genes; 29,475 bp, 9.94%), three rRNAs (5,216 bp, 1.76%), and 16 tRNAs (1,200 bp, 0.40%) (**Table 1**). Among all annotated genes, only *trnfM*-*CAU* was present in two copies, whereas all remaining genes occurred as single-copy loci. A total of 23 introns were identified in ten PCGs (*ccmFC, cox1, cox2, nad1, nad2, nad4, nad5, nad7*, *rps3* and *rps10*), including 17 *cis*-spliced and six *trans*-spliced introns, with *nad1, nad2*, and *nad5* each harboring four introns (**Supplementary Figure S2**; **Table 1**; **Supplementary Table S4**). The complete mitogenome of *A. indica* has been deposited in the NCBI Nucleotide database under accession number PP554241.

**Figure 1:**
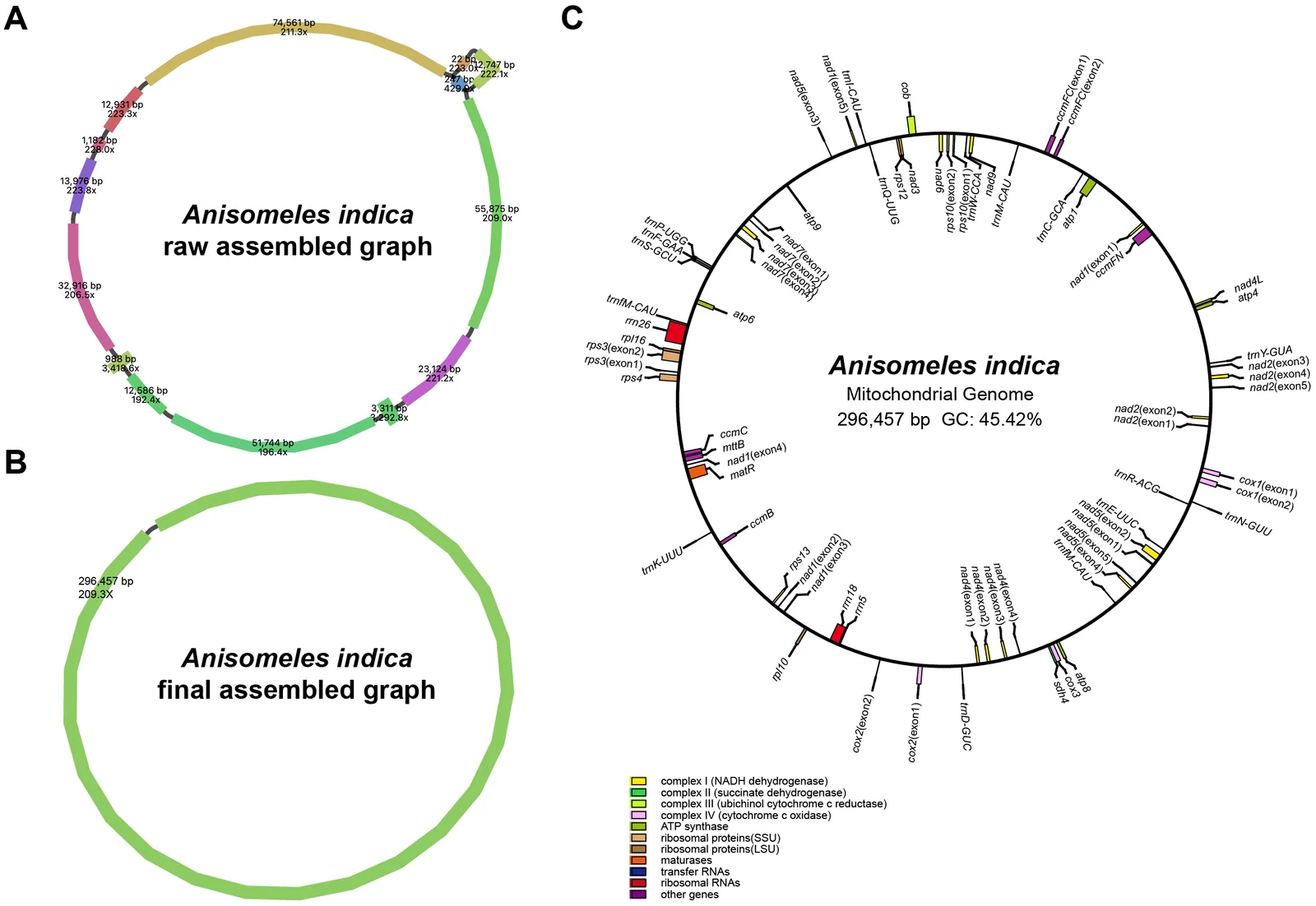
Assembly and genome organization of the *Anisomeles indica* mitogenome. **(A)** Initial assembly graph generated from HiFi reads. **(B)** Simplified assembly graph after resolving complex connections and confirming the final mitochondrial configuration. **(C)** Circular genome map of the *A. indica* mitogenome showing annotated genes and genomic features. Genes are displayed as colored blocks according to their functional categories.

**Table 1:** General characteristics of the mitochondrial genome of *Anisomeles indica*.

Genomic feature	Value
Accession number	PP554241
Length	296,457 bp
GC content	45.42%
Number of genes	51
Number of protein-coding genes	32
Number of core genes	24
Number of variable genes	8
Number of rRNA genes	3
Number of tRNA genes	16
Total length of genes (proportion)	35,891 bp (12.11%)
Total length of protein-coding genes (proportion)	29,475 bp (9.94%)
Total length of tRNA genes (proportion)	1,200 bp (0.40%)
Total length of rRNA genes (proportion)	5,216 bp (1.76%)
Number of c*is*-spliced introns	17
Number of *trans*-spliced introns	6

### 3.2 Repeat sequence analysis

A total of 60 SSRs were identified in the *A. indica* mitogenome. Tetranucleotide motifs were the most abundant repeat type (26), followed by mononucleotide (11), dinucleotide (8), trinucleotide (8), and pentanucleotide (7) repeats, whereas no hexanucleotide SSRs were detected (**Figure 2A**; **Supplementary Table S5**). Dispersed repeat analysis identified 136 repeat pairs with a minimum length of 30 bp and a cumulative repeat length of 6,857 bp (**Supplementary Table S6**). Most dispersed repeats (128, 94.1%) were shorter than 100 bp, while no repeats longer than 1,000 bp were detected (**Figure 2B**; **Supplementary Figure S3**). A total of 15 tandem repeats were identified, with repeat unit lengths of 13–71 bp and copy numbers of 1.9–3.4 (**Figure 2C**; **Supplementary Table S7**).

**Figure 2:**
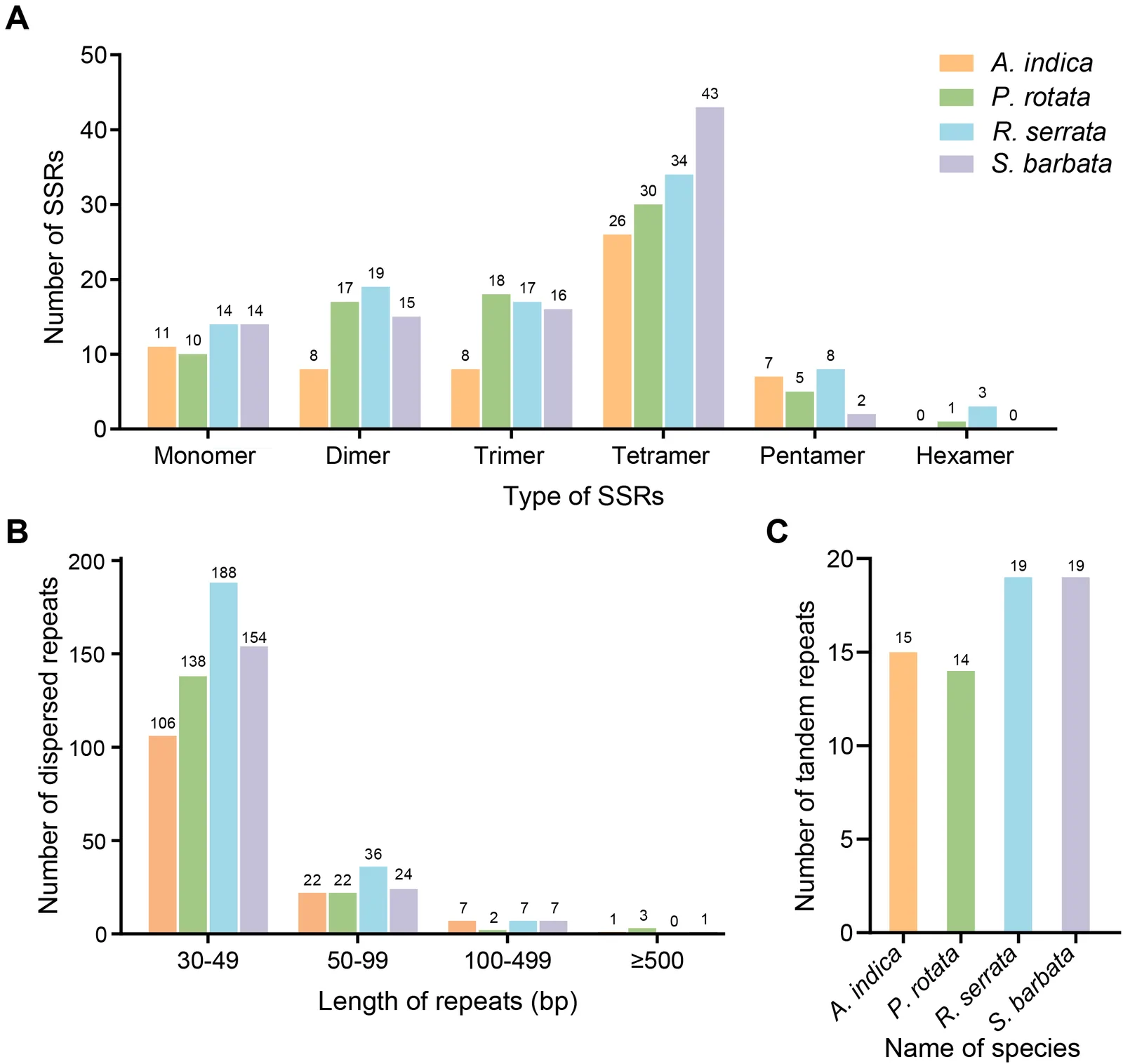
Repeat elements identified in the mitogenomes of four Lamiaceae species. **(A)** Number and composition of simple sequence repeats (SSRs) in the mitogenomes of *Anisomeles indica*, *Scutellaria barbata*, *Phlomoides rotata* and *Rotheca serrata*. **(B)** Distribution of dispersed repeats according to repeat length among the four Lamiaceae mitogenomes. **(C)** Number of tandem repeats identified in the four Lamiaceae mitogenomes.

### 3.3 Relative synonymous codon usage (RSCU) analysis

A total of 9,793 codons encoding 20 amino acids were identified in the 32 PCGs annotated in the *A. indica* mitogenome (**Supplementary Table S12**). Leucine was the most frequently encoded amino acid (1,061 codons), followed by serine (901), isoleucine (769), glycine (667), and phenylalanine (661), whereas cysteine was the least frequent (143). Codon usage in the *A. indica* mitogenome showed a clear preference for A/T-ending synonymous codons. RSCU analysis showed that 29 of the 59 synonymous codons had RSCU values greater than 1, and most of these preferred codons ended with A or T, whereas codons ending with G or C generally exhibited RSCU values below 1 (**Figure 3A**). Methionine (ATG) and tryptophan (TGG), each encoded by a single codon, had RSCU values of 1.0 (**Supplementary Table S12**; **Figure 3A**).

**Figure 3:**
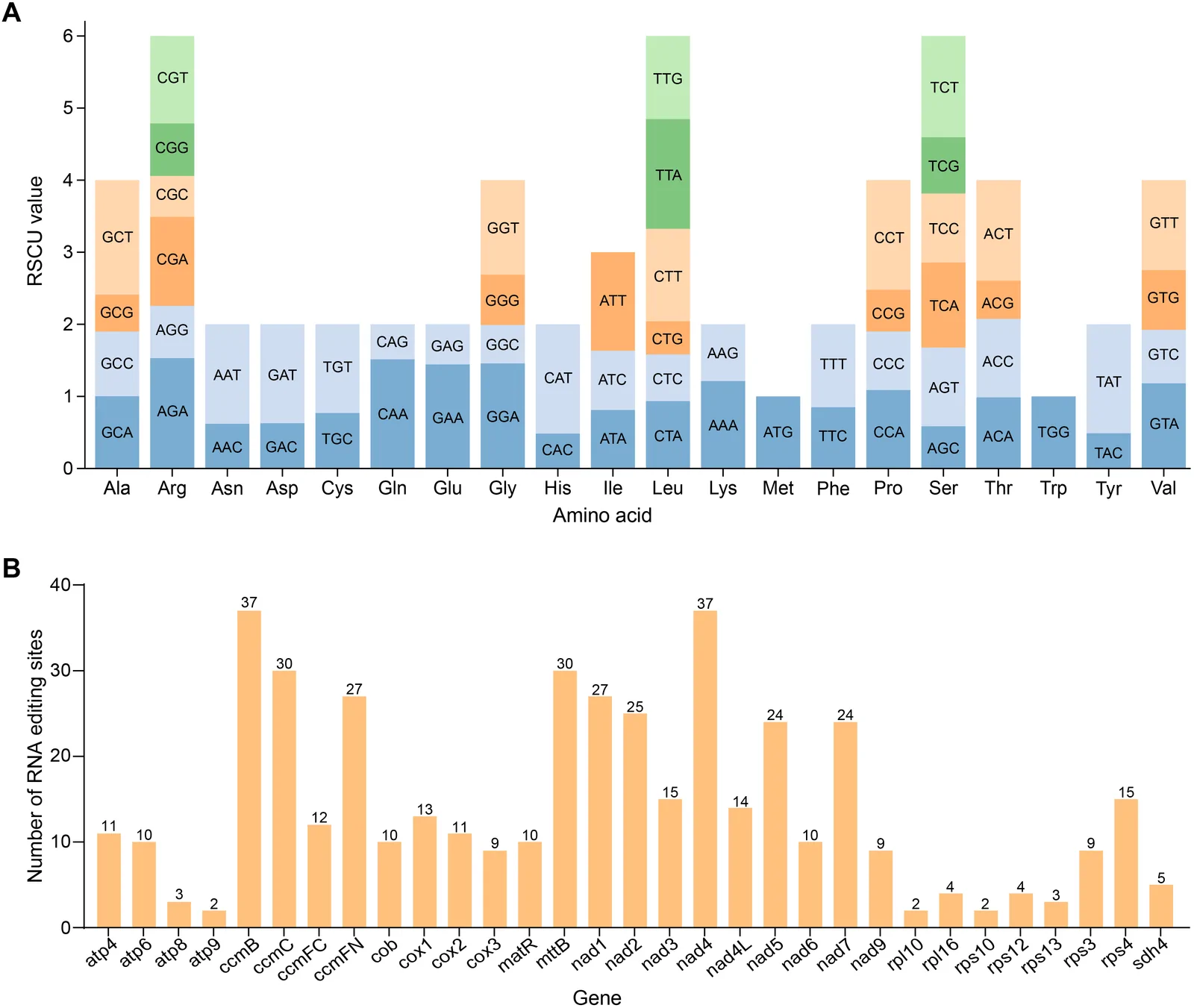
Codon usage bias and RNA editing events of the *Anisomeles indica* mitogenome. **(A)** Relative synonymous codon usage (RSCU) values of protein-coding genes in the *A. indica* mitogenome. Codons with RSCU values greater than 1 indicate preferentially used codons. **(B)** Distribution of predicted RNA editing sites across protein-coding genes in the *A. indica* mitogenome. The number of RNA editing sites predicted in each mitochondrial protein-coding gene except *atp1* is shown.

### 3.4 RNA editing analysis

RNA editing sites in the *A. indica* mitogenome were predicted using Deepred-mt, and only sites with prediction probabilities ≥ 0.90 were retained for subsequent analyses. A total of 444 putative RNA editing sites were identified across the PCGs (**Supplementary Table S13**). Predicted RNA editing sites were unevenly distributed among mitochondrial genes. No RNA editing site was predicted in *atp1*, whereas all remaining PCGs contained at least two predicted editing sites (**Figure 3B**). The highest numbers of editing sites were detected in *ccmB* and *nad4*, each harboring 37 predicted sites. In contrast, *atp9*, *rpl10*, and *rps10* each contained only two predicted editing sites. A predicted RNA editing site was identified within the start codon of *nad4L*, whereas no predicted editing events were detected within the stop codons of any PCGs (**Supplementary Table S13**). Most predicted editing events were expected to cause nonsynonymous substitutions, whereas a small proportion were synonymous (**Supplementary Table S14**). To experimentally assess the reliability of the computational predictions, 16 representative RNA editing sites were selected for validation, including six sites in *nad4*, five in *rps4*, and five in *mttB*. Comparison of genomic DNA and cDNA sequences confirmed C-to-U editing at all 16 examined sites, with C detected in genomic DNA and the corresponding T detected in cDNA (**Supplementary Figure S4**). These results provide experimental support for the predicted RNA editing events in the three selected genes.

### 3.5 MTPT analysis

A total of 30 MTPT fragments were identified in the *A. indica* mitogenome through BLASTn searches against the plastome, with a total length of 24,696 bp, accounting for 8.33% of the mitogenome and 16.26% of the plastome, respectively (**Supplementary Table S15**). The identified MTPTs ranged from 119 bp (MTPT30) to 5,298 bp (MTPT1 and MTPT2), with sequence identities ranging from 70.47% to 99.89%. The average GC content of the MTPTs was 50.05% (**Supplementary Table S15**; **Figure 4**), which was higher than the overall GC contents of both the mitogenome (45.42%) and the plastome (38.26%). The identified MTPTs collectively contained complete or partial sequences of multiple plastid genes, including the complete *rrn4.5*, *rrn5*, *rrn23*, *trnD*-*GUC*, *trnN-GUU*, *trnP-UGG*, *trnR*-*ACG*, and *trnW*-*CCA* genes, as well as partial sequences of *psaA*, *rrn16*, *trnA*-*UGC*, *trnV*-*GAC*, and *ycf1*. Importantly, the MTPT-containing regions were consistently recovered in both the PMAT and OATK assemblies, providing additional support that these plastid-derived sequences are genuine components of the *A. indica* mitogenome rather than artifacts introduced by a single assembly pipeline.

**Figure 4:**
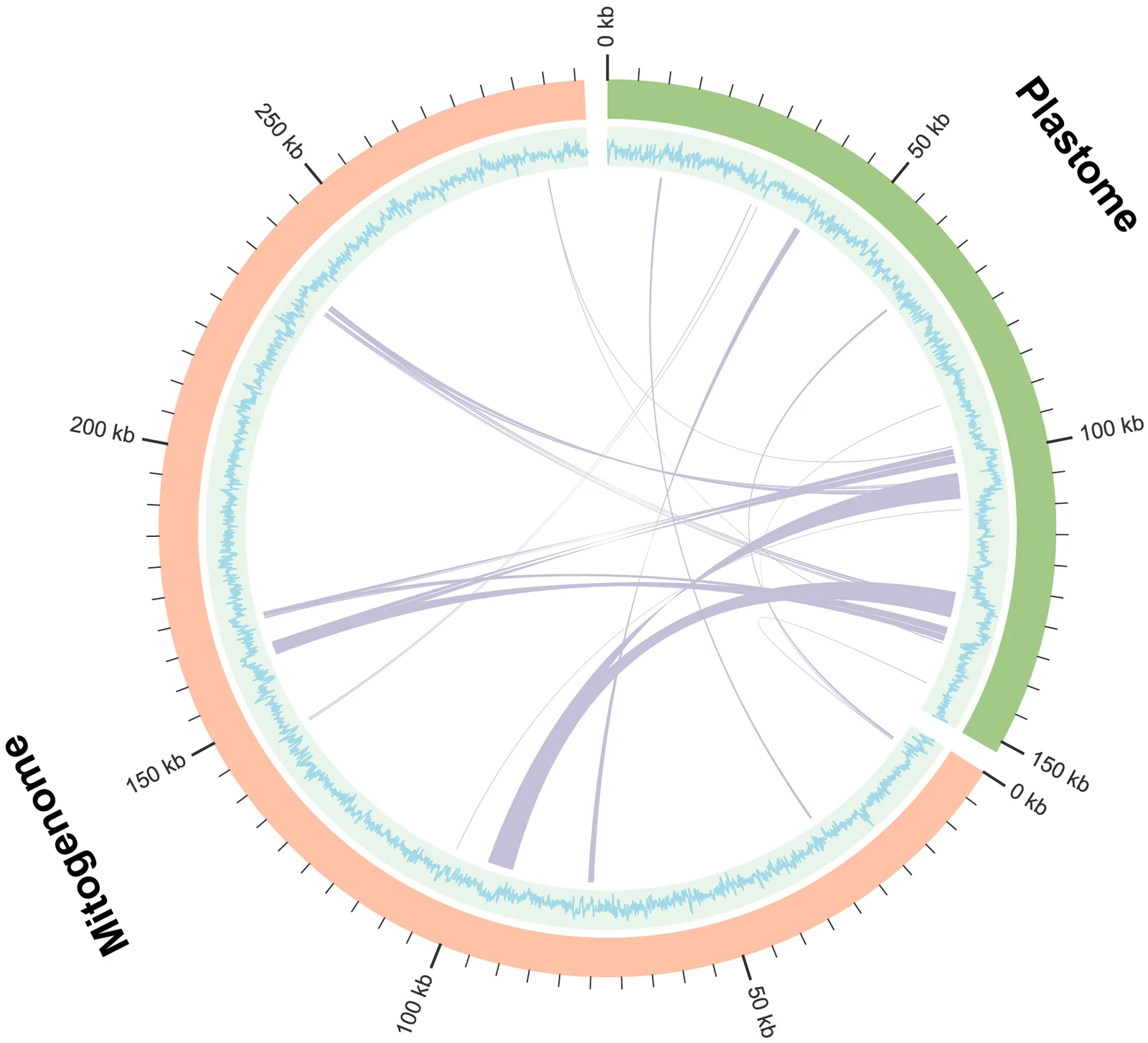
Distribution of mitochondrial plastid DNA sequences (MTPTs) in the *Anisomeles indica* mitogenome. The mitochondrial and plastid genomes are shown as orange and green arcs, respectively. The adjacent circular tracks represent the GC content of the mitochondrial and plastid genomes, while the innermost purple links indicate individual MTPT fragments transferred from the plastome to the mitogenome. Detailed information on MTPTs is provided in **Supplementary Table S15**.

### 3.6 Comparative synteny analysis

To investigate mitogenome organization and structural variation within Lamiaceae, synteny analyses were performed among the mitogenomes of *A. indica* and four representative Lamiaceae species, including *P. heyneanus*, *P. rotata*, *S. barbata*, and *R. serrata* (**Figure 5**). Among the analyzed comparisons, *A. indica* and *P. heyneanus* showed the highest level of mitogenome collinearity, with 140 syntenic blocks covering 177,276 bp and an average sequence identity of 98.10%. Similarly, *A. indica* and *P. rotata* shared 79 syntenic blocks spanning 146,396 bp with a mean sequence identity of 97.66%. In comparison, *S. barbata* and *P. heyneanus* exhibited 89 syntenic blocks with a total aligned length of 115,436 bp and an average identity of 96.24%, whereas *P. rotata* and *R. serrata* shared 97 syntenic blocks covering 122,836 bp with a mean sequence identity of 95.02% (**Supplementary Table S16**).

**Figure 5:**
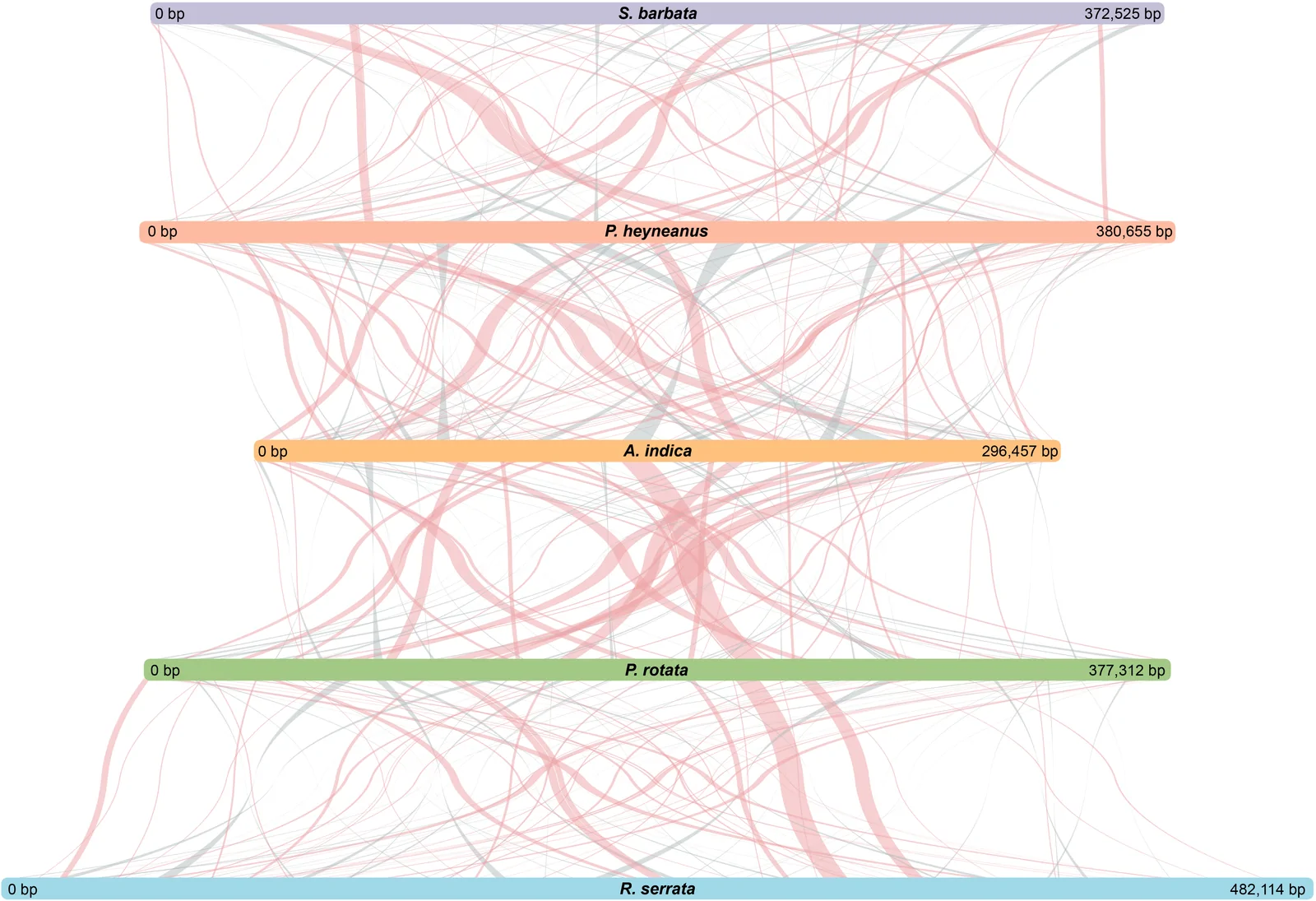
Whole-mitochondrial genome collinearity analysis of five Lamiaceae species. Collinearity analysis was performed among the mitochondrial genomes of *Anisomeles indica*, *Phlomoides rotata*, *Pogostemon heyneanus*, *Rotheca serrata* and *Scutellaria barbata*. Different colors represent distinct mitogenomes. Colored blocks between mitogenomes indicate homologous collinear regions, with pink blocks representing the syntenic regions in the same orientation and gray blocks indicating the inverted syntenic regions. .

### 3.7 Phylogenetic analysis

To infer the phylogenetic placement of *A. indica*, ML phylogenetic trees were reconstructed separately using 26 shared mitochondrial genes and 52 common plastid genes from 24 representative species, with *C. annuum* and *N. tabacum* designated as outgroups (**Supplementary Table S3**). Both ML trees exhibited largely congruent topologies with strong bootstrap support (BS) across most internal nodes (mitogenome BS score ≥ 84; plastome BS score ≥ 82) (**Figure 6**). In both organellar phylogenies, *A. indica* consistently clustered with species of the genus *Pogostemon* with maximal bootstrap support (BS score = 100). Specifically, *A. indica* formed a sister relationship with *P. heyneanus* in the mitogenome-based tree and with *P. septentrionalis* in the plastome-based tree. The *Anisomeles* + *Pogostemon* clade was further grouped with *P. rotata*, forming a strongly supported lineage within Lamiaceae. The *Scutellaria* clade was resolved as a closely related lineage, and *Rotheca serrata* was recovered as sister to the clade comprising *Scutellaria*, *Phlomoides*, *Anisomeles*, and *Pogostemon*. Species belonging to the same genera, including *Scutellaria*, *Salvia* and *Digitalis*, were consistently recovered as well-supported monophyletic groups while minor topological incongruence was observed in the lineage *Lavandula* (**Figure 6**). Although both organellar datasets recovered these taxa as a closely related group, their internal branching patterns differed slightly between mitogenome- and plastome-based trees. In the mitogenome tree, *Salvia plebeia* + *Salvia miltiorrhiza* and *Perilla frutescens* + *Elsholtzia blanda* formed sister lineages before clustering with *Lavandula*, whereas the plastome tree showed a slightly different arrangement within this group.

**Figure 6:**
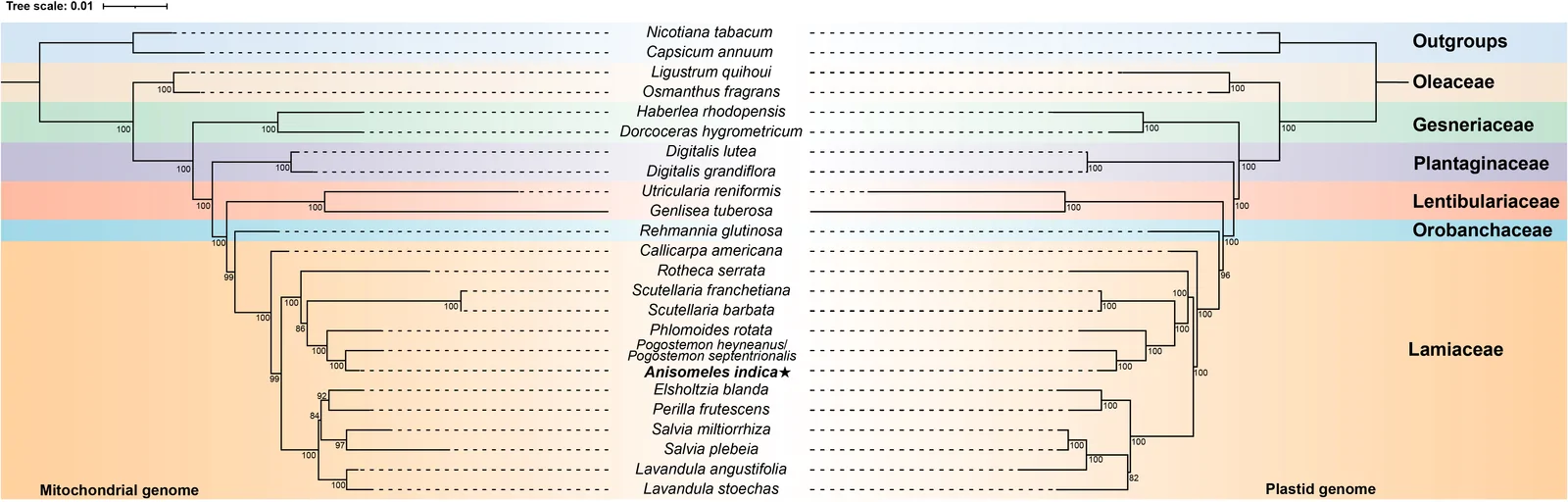
Maximum-likelihood phylogenetic trees inferred from mitochondrial (left) and plastid (right) genomes of 24 taxa in each dataset. The left and right panels represent the ML trees reconstructed based on 26 shared mitochondrial genes and 52 common plastid genes, respectively. Owing to data availability, *Pogostemon heyneanus* and *P. septentrionalis* were used as representatives of *Pogostemon* in the mitochondrial and plastid datasets, respectively. *Anisomeles indica* is highlighted in bold and marked with an asterisk. *Capsicum annuum* and *Nicotiana tabacum* (Solanaceae) were selected as outgroups. Ultrafast bootstrap support values (%) are shown at the nodes and different colors represent different plant families. Detailed information on the species and NCBI accession numbers used for phylogenetic analyses is provided in **Supplementary Table S3**.

## 4 Discussion

### 4.1 Diversity in mitogenome size and structure with conserved GC content in Lamiaceae

Plant mitogenomes are characterized by remarkable diversity in genome size and structural organization, representing one of the most dynamic genomic compartments among plant organelles (Han et al., 2024; Wang et al., 2024). Based on the 42 Lamiaceae mitogenomes analyzed in this study, the sizes of Lamiaceae mitogenomes exhibit substantial variation, ranging from 247,370 bp in *Salvia sclarea* to 572,614 bp in *Callicarpa nudiflora*, representing an approximately 2.3-fold difference in genome size (**Supplementary Table S1**). This extensive variation reflects the highly dynamic nature of mitogenome evolution in angiosperms, where genome expansion and contraction can occur through differential accumulation of foreign DNA, repeats, and structural rearrangements (Bi et al., 2024b). Among the 42 mitogenomes analyzed in this study, most reported mitogenomes have been assembled as single circular molecules, such as those of *Ajuga reptans*, *Perilla frutescens* and *Scutellaria barbata* (**Supplementary Table S1**). However, mitochondrial structures consisting of multiple circular or linear molecules have also been reported in several species, including *Ajuga ciliata*, *Mentha spicata* and *Salvia officinalis*, suggesting considerable structural flexibility during mitogenome evolution. In this study, the mitogenome of *A. indica* was assembled as a single circular molecule with a size of 296,457 bp, representing one of the smaller mitogenomes reported within Lamiaceae (**Supplementary Table S1**). Despite the extensive variation in mitogenome size, GC content remains relatively conserved among Lamiaceae mitogenomes. Among the 42 Lamiaceae mitogenomes analyzed in this study, GC content ranged from 43.92% in *Prunella vulgaris* (Sun et al., 2023) to 46.00% in *Holmskioldia sanguinea* (**Supplementary Table S1**). The *A. indica* mitogenome exhibits a GC content of 45.42%, placing it among the GC-rich mitogenomes within Lamiaceae. The relatively narrow range of GC content compared with the pronounced variation in genome size suggests that mitogenome expansion and contraction in Lamiaceae are not directly associated with changes in overall nucleotide composition.

### 4.2 Conserved core genes and variable gene repertoires in Lamiaceae mitogenomes

The gene repertoire of plant mitogenomes is generally more variable than that of plastomes due to frequent gene loss, duplication, and intracellular gene transfer events (Wang et al., 2024). Consistent with this evolutionary feature, the mitochondrial gene repertoire within Lamiaceae shows considerable variation among currently available mitogenomes. Across the 42 analyzed Lamiaceae species, the total number of annotated mitochondrial genes ranges from 39 genes in *M. spicata* to 68 genes in *S. miltiorrhiza* (**Supplementary Table S1**). This variation is mainly associated with lineage-specific differences in accessory gene components, particularly tRNAs and rRNAs, which are more frequently affected by gene duplication, loss, and intracellular transfer events during mitochondrial genome evolution (Zardoya, 2020; Wang et al., 2025a).

The number of PCGs also varied considerably among the Lamiaceae mitogenomes analyzed in this study (**Supplementary Table S1**). Although mitochondrial protein-coding sequences are generally conserved in angiosperms, the complement of variable PCGs can differ substantially among lineages because of recurrent gene loss and functional gene transfer to the nucleus (Adams et al., 2002; Adams and Palmer, 2003; Mower et al., 2012). Angiosperm mitogenomes typically retain a set of 24 core PCGs, most of which encode components involved in oxidative phosphorylation and cytochrome *c* biogenesis, whereas additional variable genes, particularly those encoding ribosomal proteins, are more frequently lost during evolution (Mower et al., 2012; Yang et al., 2022). Consistent with this general pattern, most Lamiaceae mitogenomes examined here retained 22–24 core mitochondrial genes, with the complete set of 24 core genes detected in the majority of species. Reduced core gene complements were observed in *S. sclarea* (20 core genes) and *V. trifolia* (18 core genes) (**Supplementary Table S1**), consistent with lineage-specific variation in mitochondrial gene retention and loss reported in angiosperms (Adams et al., 2002; Adams and Palmer, 2003) and Lamiaceae (Wu et al., 2025). The mitogenome of *A. indica* contains 51 annotated genes, including 32 PCGs, three rRNAs, and 16 tRNAs, and retains all 24 core mitochondrial PCGs. This gene composition indicates that *A. indica* retains the typical core mitochondrial gene complement found in most angiosperms despite the broader variation in mitochondrial gene repertoire observed within Lamiaceae (Mower et al., 2012; Yang et al., 2022).

### 4.3 Repeats contribute to mitochondrial genome size variation in Lamiaceae

Repeats are considered major drivers of plant mitogenome expansion, particularly dispersed repeats that mediate homologous recombination and generate structural diversity (Mower et al., 2012; Sloan et al., 2012; Gualberto and Newton, 2017). Comparative analysis of *A. indica*, *P. rotata*, *R. serrata*, and *S. barbata* revealed considerable interspecific variation in repeat abundance (**Figure 2**). Among these four species, *R. serrata* contained the highest numbers of both SSRs and dispersed repeats, whereas the numbers of tandem repeats were relatively similar among species, with *S. barbata* and *R. serrata* containing slightly more tandem repeats than *A. indica* and *P. rotata*. Mononucleotide to pentanucleotide SSRs were detected in all four mitogenomes, whereas hexanucleotide SSRs were found only in *P. rotata* and *R. serrata*. Although the total numbers of dispersed repeats differed among species, their length distributions were generally similar, with most repeats concentrated in the 30–49 bp size class and only a small number exceeding 500 bp. Notably, *P. rotata* was the only species among the four examined mitogenomes that contained dispersed repeats longer than 1,000 bp.

The broader comparison of the 42 Lamiaceae mitogenomes included in this study further demonstrated substantial variation in repeats. The number of SSRs ranged from 60 in *A. indica* to 138 in *Callicarpa nudiflora*, whereas the number of tandem repeats ranged from 8 in *Salvia petrophila* to 50 in *Ocimum basilicum* (**Supplementary Tables S1, S8, S9**). Dispersed repeats showed even greater interspecific variation, ranging from 86 repeats in *Salvia sclarea* to 466 in *Holmskioldia sanguinea*, with their cumulative lengths ranging from 4,629 bp in *Ajuga reptans* to 137,914 bp in *Platostoma chinense* (**Supplementary Tables S10**, **S11**). Species with relatively large mitogenomes generally contained greater amounts of dispersed repeats. In contrast, the relatively compact mitogenomes of *S. sclarea* (247,370 bp) and *Vitex trifolia* (274,779 bp) contained fewer dispersed repeats, with cumulative lengths of 11,314 bp and 6,063 bp, respectively. The relatively low accumulation of repeats in the *A. indica* mitogenome is consistent with its compact genome architecture and may contribute to reduced structural complexity.

These comparative patterns suggest that the accumulation of dispersed repeats may contribute to mitogenome size variation within Lamiaceae. Similar patterns have been reported in other angiosperm mitogenomes, where dispersed repeats and associated recombination activity play important roles in shaping mitogenome evolution (Gualberto and Newton, 2017; Wu et al., 2025). However, the relationship between repeat abundance and genome size was not strictly proportional, indicating that mitogenome expansion and contraction are likely influenced by multiple evolutionary processes, including intracellular DNA transfer, gene loss, and genome rearrangement, in addition to repeat accumulation.

### 4.4 Inter-organellar DNA transfer and plastid-derived sequences in the *A. indica* mitogenome

The identification of 30 MTPT fragments, spanning 24,696 bp and accounting for 8.33% of the *A. indica* mitogenome, indicates extensive historical DNA transfer events between the plastome and mitogenome. Similar plastid-derived sequences have been widely reported in angiosperm mitogenomes, supporting the view that intracellular DNA transfer is an important evolutionary process shaping plant mitogenome composition (Zhang et al., 2012; Sun et al., 2024). Compared with other reported Lamiales mitogenomes, the proportion of MTPTs in *A. indica* was higher than that observed in *Callicarpa americana* (21,464 bp, 4.30% of the mitogenome) and *Leonurus japonicus* (21,113 bp, 5.51% of the mitogenome) (Hu et al., 2025; Wu et al., 2025), but lower than that reported in *Dorcoceras hygrometricum* (also known as *Boea hygrometrica*) (53,440 bp, 10.5% of the mitogenome) (Zhang et al., 2012). The relatively high abundance of MTPTs in the compact *A. indica* mitogenome suggests that the extent of plastid-derived DNA incorporation is not necessarily correlated with overall mitogenome size, consistent with previous observations in angiosperms (Zhang et al., 2012; Wu et al., 2025).

The MTPTs in *A. indica* encompassed multiple complete or partial plastid genes, including complete copies of *rrn23*, *rrn4.5*, *rrn5*, *trnD-GUC*, *trnN-GUU*, *trnP-UGG*, *trnR-ACG*, and *trnW-CCA*, as well as partial fragments of *psaA*, *rrn16*, *trnA-UGC*, *trnV-GAC*, and *ycf1*. The preferential retention of rRNA and tRNA-related sequences among MTPTs has also been observed in other angiosperm mitogenomes, suggesting that highly conserved plastid regions are frequently transferred and maintained after integration into mitogenomes (Zhang et al., 2012; Yu et al., 2020; Yang et al., 2022; Zeng et al., 2024; Hu et al., 2025). In particular, plastid-derived tRNAs may potentially supplement mitochondrial tRNA repertoires and contribute to the functional evolution of organellar translation systems, as reported in several plant species including *L. japonicus* and *S. miltiorrhiza* (Yang et al., 2022; Hu et al., 2025). Interestingly, the average GC content of *A. indica* MTPT sequences was 50.05%, which was higher than that of both the mitogenome (45.42%) and plastome (38.26%). Notably, several MTPTs were derived from GC-rich plastid regions, particularly rRNA and tRNA-related regions, which may partially contribute to the higher GC content observed in *A. indica* MTPTs.

### 4.5 Phylogenetic implications of plastome and mitogenome data in Lamiales

*A. indica* was consistently recovered as the sister lineage of *Pogostemon* species in both organellar phylogenies. In the mitogenome-based tree, *A. indica* clustered with *P. heyneanus* (BS = 100), whereas in the plastome-based tree, *P. septentrionalis* was used as the representative of the genus due to the absence of available plastome data for *P. heyneanus*, and it also formed a strongly supported sister relationship with *A. indica* (BS = 100). The *Anisomeles* + *Pogostemon* lineage was further grouped with *P. rotata*, suggesting a close evolutionary relationship among these genera. Previous plastome-based studies have suggested close affinities between *Anisomeles* and members of the genus *Pogostemon* (Yao et al., 2019), and our mitogenome-based evidence provides additional support for this relationship using an independent organellar dataset. Nuclear DNA evidence generally supports this phylogenetic relationship. Previous analyses based on nuclear ribosomal internal transcribed spacer sequences supported a sister relationship between *Anisomeles* and *Pogostemon* (Yao et al., 2016; Zhao et al., 2021), while data from low-copy nuclear genes encoding pentatricopeptide repeat proteins also placed the two genera within the same closely related lineage of Pogostemoneae (Roy and Lindqvist, 2015). Nevertheless, broader sampling of Anisomeles mitogenomes will be valuable for further testing the phylogenetic placement of the genus.

Although different *Pogostemon* species were used in the mitochondrial and plastid datasets due to the limited availability of complete organellar genomes, both analyses supported a close phylogenetic relationship between *Anisomeles* and *Pogostemon*. However, this difference in taxon sampling limits direct comparisons of the two tree topologies. Despite the extensive structural rearrangements, repeat-mediated recombination, and genome size variation characteristic of plant mitogenomes, conserved mitochondrial PCGs can retain informative phylogenetic signals, as demonstrated in previous Lamiaceae studies (Hu et al., 2025; Wu et al., 2025). Integrating mitochondrial and plastid genomic information with available nuclear evidence can therefore provide complementary insights into evolutionary relationships within Lamiales. By providing the first complete mitogenome of *Anisomeles*, this study offers a valuable genomic resource for future comparative analyses, although broader taxonomic sampling is needed to determine whether the observed genomic characteristics are representative of the genus. Furthermore, although 16 representative RNA editing sites were experimentally validated, the remaining predicted sites require further confirmation, and PCR validation of the MTPT loci was not performed.

## 5 Conclusion

In this study, we assembled and characterized the first complete mitogenome of *A. indica*, representing the first mitochondrial genomic resource for the genus *Anisomeles*. The 296,457-bp mitogenome was represented as a single master circular molecule and contained 51 annotated genes. Analyses of repeats, plastid-derived fragments, and predicted RNA-editing sites revealed several important features of its mitogenome organization and evolution. In particular, the 30 identified MTPT fragments accounted for 8.33% of the mitogenome, indicating that inter-organelle sequence transfer has contributed substantially to its genomic composition. Comparative analyses identified conserved syntenic regions among the sampled Lamiaceae mitogenomes, whereas phylogenetic analyses of mitochondrial and plastid genome datasets provided congruent support for the placement of *A. indica* in a clade with *Pogostemon* and *Phlomoides*. Overall, this study expands the mitochondrial genomic resources available for Lamiaceae and provides a foundation for future investigations of mitogenome evolution and phylogenetic relationships in *Anisomeles*. Further sampling of additional *Anisomeles* species will be necessary to clarify mitogenome structural variation and evolutionary patterns across the genus.

## Data Availability

The datasets presented in this study can be found in online repositories. The names of the repository/repositories and accession number(s) can be found in the article/**Supplementary Material**.

## Supplementary material

Supplementary Figure 1 PacBio HiFi read-based validation of the mitochondrial genome configuration of *Anisomeles indica*. **(A)** Original assembly graph showing the repetitive node R1997 and its alternative connections. Node labels indicate the node ID, sequence length, and assembly depth. **(B)** Workflow used for HiFi read-based junction validation. Candidate junction sequences representing the master-circle and alternative two-circle configurations were constructed separately, and PacBio HiFi reads were remapped to each candidate junction. Only reads continuously spanning R1997 together with 2 kb of unique flanking sequence on each side and showing ≥99% sequence identity were counted as junction-supporting reads. **(C)** Master-circle configuration and IGV visualization of the two diagnostic junctions. The two junctions were supported by 167 and 145 HiFi reads, respectively. **(D)** Alternative two-circle configuration and IGV visualization of the corresponding diagnostic junctions, each of which was supported by only 5 HiFi reads.

Supplementary Figure 2 Distribution of introns in the mitogenome of *Anisomeles indica*. The left panel shows mitochondrial genes containing only *cis*-splicing introns, while the right panel displays genes containing *trans*-splicing introns. Gene names are indicated above each rectangular block.

Supplementary Figure 3 Circular map of the distribution of dispersed repeats in the *Anisomeles indica* mitogenome. The outer orange arc represents the complete mitogenome of *A. indica*. The inner circular track indicates the GC content distribution across the mitogenome, while the innermost links represent individual dispersed repeat sequences identified in the mitochondrial genome.

Supplementary Figure 4 Experimental validation of predicted mitochondrial RNA editing sites in *Anisomeles indica*. Sanger sequencing chromatograms of genomic DNA (gDNA) and complementary DNA (cDNA) showing 16 validated C-to-U RNA editing sites in *nad4* (six sites), *rps4* (five sites), and *mttB* (five sites). The nucleotide positions are indicated above the chromatograms. At each examined site, the genomic DNA contained C, whereas the corresponding cDNA contained T, confirming the predicted RNA editing events.
